# Safety and efficacy of tinea pedis and onychomycosis treatment in people with diabetes: a systematic review

**DOI:** 10.1186/1757-1146-4-26

**Published:** 2011-12-04

**Authors:** Lisa Matricciani, Kerwin Talbot, Sara Jones

**Affiliations:** 1 School of Health Sciences, University of South Australia, North Terrace, Adelaide, South Australia, 5000, Australia

**Keywords:** Onychomycosis, tinea pedis, diabetes, treatment, safety, efficacy

## Abstract

**Background:**

Effective treatment of tinea pedis and onychomycosis is crucial for patients with diabetes as these infections may lead to foot ulcers and secondary bacterial infections resulting in eventual lower limb amputation. Although numerous studies have assessed the effectiveness of antifungal drug and treatment regimens, most exclude patients with diabetes and examine otherwise healthy individuals. While these studies are useful, results cannot necessarily be extrapolated to patients with diabetes. The purpose of this study was to therefore identify the best evidence-based treatment interventions for tinea pedis or onychomycosis in people with diabetes.

**Methods:**

The question for this systemic review was: 'what evidence is there for the safety and/or efficacy of *all *treatment interventions for adults with tinea pedis and/or onychomycosis in people with diabetes'? A systematic literature search of four electronic databases (Scopus, EbscoHost, Ovid, Web of Science) was undertaken (6/1/11). The primary outcome measure for safety was self-reported adverse events likely to be drug-related, while the primary outcome measures assessed for 'efficacy' were mycological, clinical and complete cure.

**Results:**

The systematic review identified six studies that examined the safety and/or efficacy of treatment interventions for onychomycosis in people with diabetes. No studies were identified that examined treatment for tinea pedis. Of the studies identified, two were randomised controlled trials (RCTs) and four were case series. Based on the best available evidence identified, it can be suggested that oral terbinafine is as safe and effective as oral itraconazole therapy for the treatment of onychomycosis in people with diabetes. However, efficacy results were found to be poor.

**Conclusions:**

This review indicates that there is good evidence (Level II) to suggest oral terbinafine is as safe and effective as itraconazole therapy for the treatment of onychomycosis in people with diabetes. Further research is needed to establish the evidence for other treatment modalities and treatment for tinea pedis for people with diabetes. Future efforts are needed to improve the efficacy of treatment intervention.

## Introduction

Diabetes affects approximately 285 million people worldwide, with estimates expected to rise to 438 million in 2030 [1]. Diabetes is associated with a number of serious and costly health complications, in particular, diabetic foot ulcers [2,3]. Foot ulcers cause considerable disability [4,5], morbidity [6] and are the leading cause of foot amputations and hospitalisations among people with diabetes [7-11]. While measures such as foot care and patient education are acknowledged as effective strategies to prevent foot ulcers [12,13], the importance of treating tinea pedis and onychomycosis (fungal infections of the foot and toenails) is becoming increasingly recognised [14,15] with evidence to suggest that tinea pedis and onychomycosis are significant predictors in the development of foot ulcers [16]. This is particularly concerning for people with diabetes, who are 2.5 to 2.8 times more likely to have these conditions (i.e. tinea pedis and onychomycosis) than otherwise healthy individuals [17].

Both tinea pedis and onychomycosis may lead to the development of foot ulcers. Onychomycosis may result in foot ulceration as a result of a thick, sharp, brittle piece of nail piercing the skin, or as a result of vascular compromise arising from increased subungal pressure due to enlarged dystrophic nails [15,18]. Tinea pedis may also result in the formation of foot ulcers through the development of fissures in the plantar and/or interdigital skin. In both cases, injury creates a portal of entry for pathogens which promotes the development of further complications including cellulitis, osteomyelitis, gangrene and lower limb amputation. While the association between foot ulceration and the presence of tinea pedis and/or onychomycosis has not been formally tested, evidence [19-23] exists to suggest that patients with cellulitis, osteomyelitis and gangrene are also likely to have tinea pedis and/or onychomycosis. Patients with diabetes are at an increased risk of developing these complications as they often present with an impaired ability to detect injury as a result of peripheral neuropathy, retinopathy and also obesity, which may inhibits foot inspection [24,25]; as well as an impaired ability to fight infection due to elevated blood glucose levels and altered immune function [26]. Studies [24,27] also indicate that patients with diabetes and onychomycosis have a significantly higher rate of foot ulceration, gangrene and a combination of foot ulcer and gangrene compared to diabetic patients without onychomycosis. Effective and safe treatment of tinea pedis and onychomycosis is therefore especially important for patients with diabetes as it may prevent ulcer formation and secondary complications [28].

While the importance of effective and safe treatment of tinea pedis and onychomycosis in people with diabetes has been emphasised in the literature, many studies have aimed to determine the efficacy and safety of antifungal treatment interventions in otherwise healthy individuals. While these studies are of importance, results cannot necessarily be extrapolated to patients with diabetes. Firstly, people with diabetes often present with polypharmacy, many of which have the potential to interact with antifungal medication [29]. Secondly, people with diabetes tend to be resistant to treatment as high blood glucose levels and an inability to keep feet clean and dry due to obesity and/or retinopathy fosters fungal growth [30].

Many studies acknowledge the complexity of treating tinea pedis and onychomycosis in people with diabetes and provide recommendations for safe and effective treatment [14,18,30-36]. To date, there do not appear to be any studies that provide a comprehensive evidence-based review of the evidence for the safety and efficacy of different treatment interventions for tinea pedis and/or onychomycosis in people with diabetes. The purpose of this study was therefore, to determine the evidence available for the safety and efficacy of *all *treatment interventions and modalities for tinea pedis and onychomycosis in patients with diabetes.

## Methods

A systematic literature search was conducted to determine the evidence for all antifungal treatment interventions for adults (aged 18 years and older) with tinea pedis and/or onychomycosis of the toenails in people with diabetes.

### Criteria for inclusion

Any study that examined the safety and/or efficacy of a treatment intervention for tinea pedis and/or onychomycosis of the toenails in adults with Type 1 or Type 2 diabetes was considered for inclusion in this review. Only studies that used microscopy and culture to establish the presence of fungal infections were included. The primary outcome measures assessed in this review were 'safety' and 'efficacy'. 'Safety' was defined as any self-reported adverse events likely to be related to the treatment intervention, while 'efficacy' included measures of *mycological cure*, defined as negative potassium hydroxide culture; *clinical cure*, defined as less than 10% nail involvement and *complete cure*, which was defined as complete mycological and clinical cure. Studies that did not provide sufficient detail of the diagnosis, treatment regimen or the outcome variables were excluded from this review, as were studies that examined onychomycosis of the hands. Two reviewers (LM and KT) independently applied these criteria to locate trials for this review.

### Systematic search strategy

Four electronic databases (Scopus, EbscoHost, Ovid, Web of Science) were searched using the search strategy summarised in Table 1. A preliminary search determined the scope and relevance of candidate databases. All abstracts were screened for inclusion criteria. Potentially eligible papers were read in full and all relevant papers were kept and included in this review. No date or language limits were set. The last search was carried out was on January 6 2011. Reference lists of all eligible papers were reviewed to locate any additional studies. No date limits were set, however, only papers written in English were considered for inclusion.

**Table 1:** Search strategy used for each database.

Database	Date	Limitation	Search Terms
Scopus	15/10/10	Abstract	((ABS("Tinea Pedis" OR "onychomycosis" OR "athlete's foot"))) AND ((ABS(diab*)) AND (ABS(treat*)).
Ovid	6/1/11	Abstract	((Tinea Pedis OR onychomycosis OR athlete's foot) and (treat$) and (diab$)
Web of Science	6/1/11	Topic	Topic = ("Tinea Pedis" OR "onychomycosis" OR "athlete's foot") AND Topic = (diab*) AND Topic = (treat*)
EbscoHost	15/10/10	Abstract	AB ("Tinea Pedis" OR "onychomycosis" OR "athlete's foot") and AB (diab*) and AB (treat*)

### Evaluation, analysis and synthesis of studies included for review

All studies included in this review were read by two reviewers independently (LM and KT) for content extraction and appraised for their level of evidence using the NHMRC hierarchy of evidence scale [37] (Table 2). Each study was then independently evaluated for its internal and external validity and rated according to the scale [38] provided by the American Occupational Therapy Association's (AOTA) Evidence-Based Practice Project (Table 3). Specific threats to validity were also identified and recorded. All studies were assessed by two independent reviewers (LM and KT) and any discrepancies were resolved by a third independent reviewer (SJ).

**Table 2:** NHMRC Hierarchy of evidence

Level of evidence	Study design
I*	A systematic review of level II studies
II	A randomised controlled trial
III-1	A pseudorandomised controlled trial
III-2	A comparative study with concurrent controls
III-3	A comparative study without concurrent controls
IV	Case series with either post-test or pre-test/post-test outcomes

**Table 3:** Levels of evidence for the AOTA Evidence-Based Practice Project

Level	Definition
Design	
I	Randomized trial: Comparison of two or more groups or conditions in an experiment with random assignment to group or to sequence of conditions in a repeated-measures design
II	Non-RCT: Comparison of two or more groups or treatments in a quasi-experiment without randomization to group, condition, or sequence
III	Non-RCT: Comparison of one group pre- and post-treatment
IV	Single-subject design: One subject measured at intervals throughout an intervention
N	A Narratives and case studies
Sample size	
A	*n *≥ 50 persons per condition or group or observations in a single subject design
B	*n *≥ 20 persons per condition or group or observations in a single subject design
C	*n *< 20 persons per condition or group or observations in a single subject design
Internal validity	
1	High internal validity: No strong alternate explanation for outcome *or *other threats to validity, such as attrition, unblinded evaluation, unequal treatment, or spontaneous recovery
2	Moderate internal validity: No strong alternate explanation for outcome but one or two threats to validity exist
3	Low internal validity: Does not meet criteria for 1 or 2
External validity	
a	High external validity: Participants represent population *and *treatment represents current practice *or *has strong theoretical support *and *the research was done in a natural (home or clinic) setting
b	b Moderate external validity: Has two of the criteria listed for a
**c**	Low external validity: Has one or fewer criteria listed for a

AOTA-American Occupational Therapy association; RCT-Randomised controlled trial. Figure taken from Tromblay and Ma [38]

The methodological quality of all randomized controlled trials (RCTs) included in this review were further appraised using the PEDro scale. The PEDro scale has established reliability [39] and provides a score out of 11 which provides a measure of the validity of a study's conclusions. Question 1 relates to the external validity, questions 2-9 relates to internal validity and questions 10-11 relates to whether the study contains sufficient statistical information to make the results interpretable. These articles were also assessed by two independent (LM and KT) reviewers and any discrepancies were resolved by a third independent reviewer (SJ).

## Results

**Figure 1:**
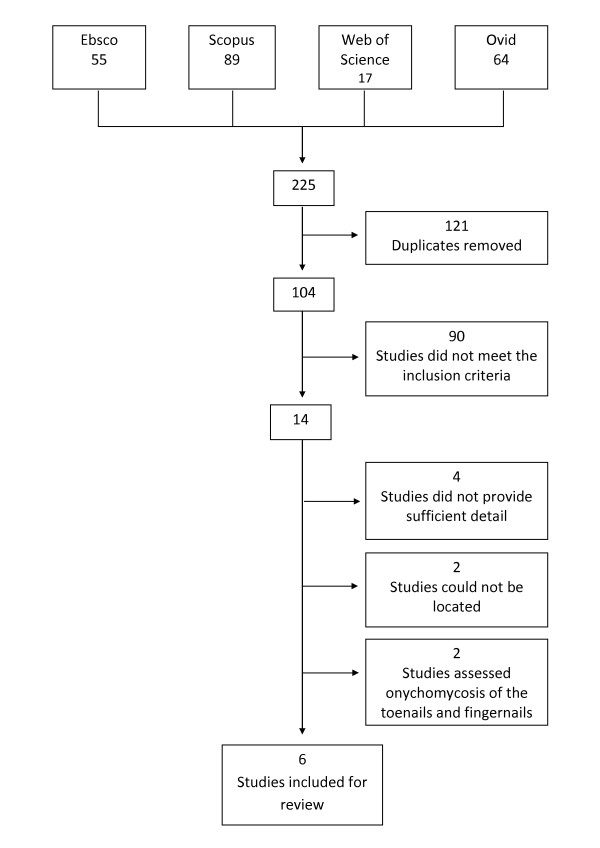
**Modified flow chart of search strategy**.

The systematic review identified 14 different studies that examined the efficacy and/or safety of treatment interventions for onychomycosis and/or tinea pedis in people with diabetes (Figure 1). Of these, four [19,40-42] were excluded because they did not provide sufficient detail about the diagnosis of infection and/or the outcome variables assessed and two [43,44] were excluded because they assessed treatments interventions for onychomycosis of the toenails and fingernails and did provide results for the two separate conditions. A further two [45,46] studies could not be included because an abstract or full text copy of the study could not be located, despite efforts to contact the authors and searching overseas libraries. Thus, a total of six different studies [47-52] were in included in this review.

Of the six [47-52] studies included in this review, two [50,51] examined safety, one [47] examined efficacy and three [48,49,52] examined both the safety and efficacy of a treatment intervention (Table 4). All studies examined treatment for onychomycosis. None examined treatment for tinea pedis. While we searched for both pharmacological and non-pharmacological antifungal treatment interventions, only pharmacological interventions were assessed in trials that determined efficacy. The same interventions were also examined in trials that assessed safety, one [51] study also examined palliative treatment, which consisted of toenail trimming and cleaning. The pharmacological interventions examined included continuous oral terbinafine therapy, pulse oral itraconazole therapy and daily topical ciclopirox 8% therapy with mechanical debridement every eight weeks. There were no trials examining alternative medical therapies.

**Table 4:** Summary of studies included for review

Study [ref]	Design	Subjects n (males)	Age Mean (SD)	Intervention	Assessment (weeks)	Outcome measures Safety	Outcome measures Efficacy
Pollak [44]	Case series	77 (N/R)	N/R	**Active Intervention: **250 mg oral terbinafine once daily for 12 weeks	0, 6, 12, 18, 24, 30, 36, 48, 72	Participant and investigator-reported adverse events. Severity (mild, moderate, severe, life-threatening) and likelihood of adverse event related to treatment (yes, no, uncertain) determined by investigator	Not assessed
				**Control **: no control			
Albreski [45]	RCT	52 (51)	71.42 (6.21) years	**Active Intervention: **200 mg of oral itraconazole twice daily for one week over three consecutive months	0, 2, 10, 32	Investigator-reported adverse events and likelihood that any adverse events were related to drug therapy	Not assessed
				**Control**: Toenail trimming, cleaning and soaking for 4 months.			
Brenner [43]	Case series	49 (36)	63.8 (12.0)years	**Active Intervention: **Daily topical application of Ciclopirox 8% nail lacquer with mechanical debridement every eight weeks for 48 weeks.	0, 8, 16, 24, 32, 40, 48	Participant and investigator-reported adverse events. Severity and likelihood of adverse event related to treatment determined by investigator.	*Mycological cure*: negative results on microscopy (KOH) and fungal culture. *Clinical cure*: ≥ 90% improvement (from baseline) in diseased nail. *Complete cure*: mycological cure plus clinical cure.
				**Control**: no control			
Farkas [46]	Case series	89 (47)	55.7 (11.7) years	**Active intervention: **250 mg oral terbinafine once daily for 12 weeks.	0, 4, 8, 12, 24, 36, 48	Self-reported adverse event (severity was scored as mild, moderate or severe) and the likelihood of any adverse events being related to the intervention (non, unlikely, possible, probably, certain)	*Mycological cure*: negative results on microscopy and fungal culture of samples taken from the target toe nail. *Clinical cure*: 100% clearing of the target toenail. *Complete cure*: mycological cure plus clinical cure
				**Control: **no control			
Gupta [42]	RCT	70 (34)	60.67 (1.52) years	**Active intervention: **200 mg oral itraconazole twice daily for one week of three consecutive months	0, 1, 6, 12,24, 36, 48, 60, 72	Self-reported adverse event and the likelihood of any adverse events being related to the intervention	*Mycological cure*: negative results on microscopy and fungal culture. *Clinical cure*: ≤ 10% nail plate involvement. *Effective cure*: mycological cure plus either clinical cure.
				**Control: **250 mg oral terbinafine daily for 12 weeks.			
Sadighha [41]	Case series	13 (N/R)	50-73 years	**Active intervention: **200 mg oral itraconazole twice daily for one week of four consecutive months	0, 26	Not assessed	*Complete cure*: negative mycological culture and resolution of nail deformity.
				**Control**: no control			

RCT = Randomised controlled trial

N/R = not reported

all studies, except that by Farkas and colleagues [46] assessed people with Type 2 diabetes. Farkas and colleagues did not find any significant differences in the treatment outcomes for patients with Type 1 and Type 2 diabetes, the pooled results from this study were considered in this review

As shown in Table 5, two [48,51] studies provided level II evidence [randomised controlled trials (RCT)] and four [47,49,50,52] studies provided level IV evidence (case series). Internal validity varied across studies. Of the six studies, one [48] scored 'high', three [47,50,52] scored 'moderate' and two [49,51] scored 'low' for internal validity. Common threats to internal validity included a lack of blinding and attrition. External validity scores also varied across studies, but to a lesser extent. In total, two studies were found to have high external validity and four studies were found to have moderate external validity. Common threats to external validity included the inclusion of participants who were not truly representative of people with diabetes. All studies were limited by a small sample size.

**Table 5:** Appraisal of the validity of the studies included for review using the AOTA scale

Study	Level of Evidence^†^	Sample size	Internal validity	Possible threats to internal validity	External validity	Possible threats to external validity
Pollak [44]	IV	A	2-Moderate	Unblinded	B-Moderate	*Treatment may not represents current practice:* - Participants had to have toenails that the investigators believed were capable of regrowth - Onychomycosis must have been dermatophyte-caused - *Patients may not be representative of the diabetic population:* - Patients were not allowed to participants if they had abnormal laboratory results - Detail of diabetic population not provided (age, sex ratio, coexisting medical conditions, use of other medications etc.
Albreski [45]	II	B	3-Low	Unblinded Duration of diabetes different between groups More people in the itraconazole group received insulin compared with palliative group	B-Moderate	*Patients may not be representative of the diabetic population:* - Participants were excluded if they were taking certain medication, such as medication for high cholesterol, which patients with diabetes are likely to be on - Only one female assessed
Brenner [43]	IV	B	3-Low	Unblinded Attrition Patients were allowed to use other antifungal agents for coexisting tinea pedis Patients received also received nail care treatment	B-Moderate	*Patients may not be representative of the diabetic population:* - Patients had to have a good history of scheduled podiatric medical visits for nail care, be in good general health have good pulses.
Farkas [46]	IV	A	2-Moderate	Unblinded Attrition	A-High	nil
Gupta [42]	II	B	1-High	nil	A-High	nil
Sadighha [41]	IV	C	2-Moderate	Unblinded Selection criteria not clearly stated	B-Moderate	*Patients may not be representative of the diabetic population:* - Detail of diabetic population not provided (age, sex ratio, coexisting medical conditions, use of other medications etc.)

**^† ^**For consistency, the NHMRC study design classification system was used instead of the AOTA study design classification system

Table 6 presents the PEDro scores for the two RCTs included in this review. As shown, Albreski and colleagues [51], scored much lower than Gupta and colleagues [48] due to blinding and participant allocations.

**Table 6:** PEDro scores for the RCTs included in this study

	Eligibility criteria specified	Random allocation	Concealed allocation	Baseline comparability	Blind subjects	Blind therapists	Blind assessors	Adequate follow-up	Intention to treat analysis	Between group comparisons	Point estimates and variability	PEDro Score (/11)
Albreski [45]	Y	Y	N	N	N	N	N	Y	Y	Y	N	**5**
Gupta [42]	Y	Y	Y	Y	N	Y	Y	Y	Y	Y	N	**9**

### Safety of antifungal treatment interventions in diabetic patients

A total of five [48-52] different studies examined the safety of treatment interventions for onychomycosis in people with diabetes. Of these, two [48,51], were randomised controlled trials and three [49,50,52] were case series. Internal validity was considered 'high' for only one [48] study, while external validity was considered 'high' for two [48,52] studies.

Table 7 presents the results of the five studies that examined the safety of treatment interventions. As shown, all studies reported few or no adverse events that were likely to have been related to the treatment. None of the studies reported any life threatening adverse events that were likely to be due to treatment. The most common reported adverse event was gastrointestinal pain, which was reported for both terbinafine and itraconazole therapy. The safest interventions were palliative care and oral terbinafine therapy, which, according to Albreski and colleagues [51] and Gupta and colleagues [48] (respectively), did not produce any adverse events. The greatest number of adverse events were reported in the study by Brenner and colleagues [49], who investigated the safety of topical ciclopirox 8% with mechanical nail debridement every eight weeks. In this study, adverse events likely to be due to treatment occurred in 29% (14/49) of participants.

**Table 7:** safety outcome results identified in studies included for review

Study	Design	Treatment	Treatment regimen	Sample size (males)	Age [mean (SD)] years	Safety assessment (week)	% of patients experienced to adverse events	% of patients experienced to adverse events due to treatment intervention	Reported adverse events likely to be related to treatment intervention
Pollak [44]	Case series	Terbinafine	250 mg oral terbinafine once daily for 12 weeks†	77 (N/R)	N/R	6, 12,18, 24, 30, 36, 48, 72	61% (47/77)	10.4% (8/77)	Gastrointestinal
Albreski [45]	RCT	Itraconazole Palliative	200 mg of oral itraconazole taken twice a day for the first week of three consecutive months Toenail trimming, cleaning and soaking^†^	27 (26) 25 (25)	70.52 (7.99) 72.32 (4.42)	32 32	15% (4/27) 0%	4% (1/27) 0%	Elevated liver function test N/A
Brenner [43]	Case series	Ciclopirox 8%	Ciclopirox 8% nail lacquer applied daily to nail and 5 mm surrounding skin for 48 weeks. Nails care every 8 weeks.	49(36)	63.8 (12.0)	48	44.9% (22/49)	29% (14/49)	Toenail disorders and infection
Farkas [46]	Case series	Terbinafine	250 mg oral terbinafine once daily for 12 weeks.	89 (47)	55.7 (11.7)	36	13.5% (12/89)	7.9% (7/89)	Gastrointestinal disturbance, headache, change in taste sensation and
Gupta [42]	RCT	Itraconazole Terbinafine	200 mg of oral itraconazole taken twice a day for the first week of three consecutive months 250 mg oral terbinafine once daily for 12 weeks.	35 (16) 35 (18)	57.77 (2.3) 63.65 (1.9)	48 48	Not reported Not reported	8.6% (3/35) 0%	Gastrointestinal N/A

RCT-Randomised controlled trial

N/R-Not reported

N/A-Not applicable

^† ^According to Health Care Financing Administration [54] guidelines for 4 months.

Gupta and colleagues [48] provided the highest level of evidence for the safety of antifungal treatment interventions for onychomycosis in people with diabetes. In this study, there was no significant difference in the safety of oral terbinafine (0%) and itraconazole (8.6%). Thus, the best available evidence indicates continuous oral terbinafine is as safe as pulse oral itraconazole therapy for treating onychomycosis in people with diabetes.

### Efficacy of antifungal treatment interventions in diabetic patients

A total of four [47-49,52] different studies examined the efficacy of treatment interventions for onychomycosis in people with diabetes. Of these, one [48] was a RCT and three [47,49,52] were case series. Internal and external validity was found to be moderate to high in all but one[49] study.

**Figure 2:**
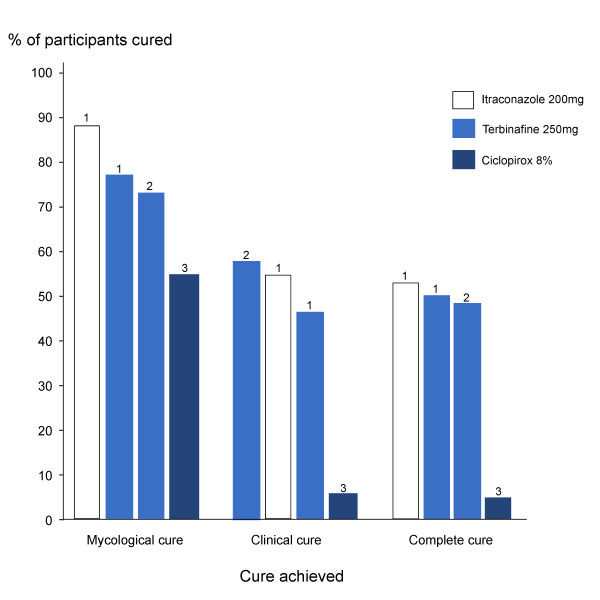
**Studies that determined the efficacy of treatment interventions**.

As shown in Figure 2 and Table 8 at week 48, mycological and complete cure was highest for oral itraconazole therapy, followed by oral terbinafine and topical ciclopirox 8% therapy. In contrast, clinical cure was slightly greater for oral terbinafine therapy than for oral itraconazole therapy. However, as shown, this was not consistent across studies. While there was slight differences amongst studies, Gupta and colleagues [48] provided the strongest evidence. In this study, there was no significant difference in the effectiveness of itraconazole and terbinafine in achieving mycological, clinical and complete cure. It can therefore be inferred that current, best available evidence suggests that pulse oral itraconazole therapy is as effective as continuous oral terbinafine therapy at achieving mycological, clinical and complete cure of onychomycosis in people with diabetes.

**Table 8:** Summary of studies that examined the safety of treatment interventions for people with diabetes

Study	Design	Subjects	Infection	Organism	Intervention	Assessment (week)
Gupta [42]	RCT	*Itraconazole* N = 35 Sex = 16 M; 19 F Age = 57.77 (2.3) yr *Terbinafine* N = 35 Sex = 18 M; 17 F Age = 63.65 (1.9) yr	DLSO	Dermatophyte	*Ciclopirox 8%* Ciclopirox 8% nail lacquer applied to nail and 5 mm surrounding skin. Daily application over the previous coat, removed after 7 days using isopropyl alcohol. Nails trimmed and debrided at each scheduled visit (8 weeks)	48

Brenner [43]	Case series	N = 49^†^ Sex = 36 M; 13 F Age = 63.8 (12.0) yr Duration of diabetes = 8.4 (7.4) yr Duration of infection = 10.8 (15.3) yr	DSO^††^	Not reported^†^	*Terbinafine* 250 mg terbinafine daily 12 weeks	48

Farkas [46]	Case series	N = 89 Sex = 47 M; 42 F Age = 55.7 (11.7) yr Duration of diabetes = 10.2 (7.7) Duration of infection = 5.4 (6.0)	DSO	Dermatophyte (67.4%); moulds (5.6%); yeast (4.5%)	*Itraconazole pulse therapy *200 mg of oral itraconazole taken twice a day for the first week of three consecutive months *Terbinafine *250 mg of oral terbinafine once daily for 12 weeks.	48

Sadighha [41]	Case series	N = 13 Sex = NR Age = NR	DSO^††^	Dermatophyte^†^	*Pulse itraconazole therapy *200 mg of oral itraconazole taken twice a day for the first week of four consecutive months	26

^† ^Concomitant tinea pedis was present.

"DSO" = distal subungual onychomycosis; "DLSO" = distal lateral subungual onychomycosis

Study 4 is excluded from the graph because efficacy was not assessed at week 48 like the rest of the studies. Sadighha [41] found itraconazole achieved cure in 7.7% (1/13) of patients with diabetes at week 26

While Brenner and colleagues [49] found daily, topical application of ciclopirox 8% to result in poor efficacy outcomes, the internal validity for this study was low and the external validity was moderate. Current available evidence is therefore unable to provide a confident indication of the efficacy of ciclopirox 8% for the treatment of onychomycosis in adults with diabetes.

## Discussion

### Main findings

This systematic literature review identified six different studies that examined the safety and/or efficacy of antifungal treatment interventions for onychomycosis of the toenails in adults with diabetes. Of these, two [48,51] were RCTs and four [47,49,50,52] were case series. Not a single study examined treatment interventions for tinea pedis. From the available studies, the strongest evidence suggests that continuous oral terbinafine therapy is as safe and effective as pulse itraconazole therapy for treating onychomycosis.

### Strengths and limitations

This is the first systematic literature review to examine the evidence for the safety and/or efficacy of treatment interventions for tinea pedis and onychomycosis of the toenails in adults with diabetes. There are however, several limitations that need to be addressed. Firstly, only studies written in English were included in this review. Secondly, since none of the studies were longitudinal, the safety and efficacy of repeated or long term use of such medications is unclear. Lastly, although this study applied strict inclusion/exclusion criteria for selecting studies to be included in this review, none of the studies explicitly stated how nail involvement was measured and calculated, limiting the reliability of comparisons made between studies that reported clinical cure.

### Safety

The safety of oral antifungal agents for people with diabetes, especially those taking insulin or oral hypoglycaemic medications has raised much interest. Itraconazole and other drugs in the imidazole family (i.e. ketoconazole, fluconazole), act as a competitive inhibitor of the cytochrome P450 (CYP) 3A4 isoenzyme and therefore have the potential to increase the risk of hypoglycemia in people with diabetes who are taking oral hypoglycaemic medication that are metabolised by this pathway [40,53]. Since many other medications are also metabolised by this pathway, itraconazole also has the potential to interact with a myriad of other medications, including HMG-CoA reductase inhibitors, calcium channel blockers, warfarin, cyclosporine, benzodiazepines and certain antiarrythmic medication. Given the nature of diabetes, it is likely that some patients would also be taking some of these medications.

While the studies included in this review suggests itraconazole "to be safe for patients with diabetes", most studies had limited external validity. Although two [48,52] studies scored 'high' for external validity, these studies examined participants who were taking oral hypoglycaemic medications that are not metabolised by the cytochrome P450 (CYP) 3A4 pathway [48]. Thus, careful consideration is still required before prescribing itraconazole for patients with diabetes.

Unlike, itraconazole, terbinafine is metabolised by the cytochrome P450 (CYP) 2D6 isoenzyme [31]. Since this enzyme is not involved in the metabolism of oral hypoglycaemic medications, terbinafine is unlikely to increase the risk of hypoglycemia in people with diabetes who are taking oral hypoglycaemic medication [48]. Terbinafine may therefore be a safe alternative for people who are unable to take itraconazole.

### Efficacy

Given the complications that may arise if tinea pedis and onychomycosis are not treated, the effectiveness of antifungal treatment interventions is of great importance. While it is often suggested that patients with diabetes are more resistant to antifungal treatment interventions than non-diabetic patients [30], the studies identified in this review presented cure rates that were comparable to people who did not have diabetes. In spite of this, the overall complete cure rate was between 7.7% and 52.9% after 48 weeks of treatment, this is a considerable length of time for a patient to be at an increased risk of developing a foot ulcer. It is also a significant number of patients who are never cured during this period of time, but who still have the expense of treatment and who have possibly incurred the unwanted side effects of the medications. A treatment intervention that achieves a greater cure rate and provides cure in a shorter time period is therefore needed for patients with diabetes.

## Conclusion

This review indicates that there is good evidence (Level II) to suggest that continuous oral terbinafine is as safe and effective as pulse oral itraconazole therapy for the treatment of onychomycosis in people with diabetes. However, efficacy results are poor. There is no evidence for the treatment of tinea pedis for people with diabetes. While there are numerous alternative and adjunctive antifungal treatment interventions, including mechanical debridement, combination therapy and podiatric intervention that have been recommended for patients with diabetes within the literature, such alternative options have never been examined. Based on the paucity of studies identified and the overall poor efficacy of examined antifungal agents, further research is needed to determine the evidence for alternative treatment modalities and to identify interventions that provide safe and more effective treatment of tinea pedis and onychomycosis in people with diabetes.

## Competing interests

The authors declare that they have no competing interests. This study received no funding

## Authors' contributions

LM carried out the systematic review and study appraisal and drafted the manuscript. KT carried out the systematic review and study appraisal. SJ assisted to locate studies of relevance for the study and assisted in the appraisal of included studies. All authors read and approved the final manuscript.
